# Could Arterial Spin Labeling Distinguish Patients in Minimally Conscious State from Patients in Vegetative State?

**DOI:** 10.3389/fneur.2018.00110

**Published:** 2018-03-02

**Authors:** Bing Wu, Yi Yang, Shuai Zhou, Wei Wang, Zizhen Wang, Gang Hu, Jianghong He, Xinhuai Wu

**Affiliations:** ^1^Department of Radiology, PLA Army General Hospital, Beijing, China; ^2^Department of Neurosurgery, PLA Army General Hospital, Beijing, China; ^3^Inner Mongolia Medical University, Hohhot, China

**Keywords:** disorders of consciousness, minimally conscious state, arterial spin labeling

## Abstract

**Purpose:**

Diagnostic error is common among patients with vegetative state (VS) and minimally conscious state (MCS). The purpose of this article is to use three-dimensional pseudo-continuous arterial spin labeling (pcASL) to compare cerebral blood flow (CBF) patterns in patients in MCS with those in VS.

**Methods:**

Patients meeting MCS and VS criteria were identified. Two post-labeling delay (PLD) time pcASL on 3.0-Tesla magnetic resonance imaging scanner system were performed with patients in the resting awake state. After registration to T1WI structure imaging, multiple brain regions of interest of ASL CBF map were automatically separated. The average CBF value of every brain region was calculated and compared between the MCS and VS groups with *t*-tests.

**Results:**

Fifteen patients with VS were identified, with ages ranging from 33 to 71 years. Eight patients who met the MCS criteria ranged in age from 23 to 61 years. Compared with VS, the regional CBF for MCS had a pattern of significantly increased CBF in the regions including the putamen, anterior cingulate gyrus, and medial frontal cortex. A left-lateralized pattern was observed to differentiate MCS from VS. CBF with PLD 2.5 s could find more regions of pattern differentiating MCS from VS than with PLD 1.5 s, except for the pallidum.

**Conclusion:**

MCS might be differentiated from VS by different ranges of regional CBF as measured by ASL. Multi-PLD ASL may serve as an adjunct method to separate MCS from VS and assess functional reserve in patients recovering from severe brain injuries.

## Introduction

A subset of coma patients develops a prolonged impairment in consciousness, such as the vegetative state (VS) and minimally conscious state (MCS). Diagnostic error is common among patients with VS and MCS (1). Because of variable behavior observed at the bedside, approximately 30–40% of people diagnosed with VS actually retain conscious awareness (2).

Arterial spin labeling (ASL) is a magnetic resonance perfusion method that measures cerebral blood flow (CBF) *in vivo*. Unlike other comparable functional imaging modalities, ASL avoids the use of a radioactive tracer or gadolinium and is noninvasive. Recent advances in magnetic resonance imaging (MRI) technology, including higher magnetic fields, array receiver coils, pseudo-continuous arterial spin labeling (pcASL) sequences, and rapid three-dimensional (3D) acquisition techniques, have made it feasible to apply ASL in a wide range of clinical applications (3–9), especially in the characterization of brain states, longitudinal follow-up, or monitoring treatment effects.

Cerebral blood flow has been demonstrated to have a strong association with neural activity (10). Preliminary studies suggest a marked reductions in cerebral metabolism in MCS patients (11), suggesting that CBF may also be decreased.

A previous study identified globally decreased CBF and a selective reduction of CBF within the frontal cortical regions as well as gray matter (GM) in MCS patients compared with normal control subjects (12). The purpose of our study was to use 3D pcASL to compare CBF patterns in MCS patients with those in VS patients.

## Materials and Methods

### Patient Selection

The study was approved by the Ethics Committee of the PLA Army General Hospital. Consent for this study was obtained for patients from a legally authorized representative. Control subjects provided their own consent. Patients who met the criteria for MCS and VS were identified through discussions with physicians in the inpatient Neurosurgery Department of the PLA Army General Hospital between 2012 and 2016. Clinical subjects were adults who had sustained stroke, traumatic brain injury, or hypoxic ischemic injury. Subjects were included if they were between 18 and 75 years of age, had non-progressive severe brain injury, were at least 1 month post-injury, and met the Aspen Consensus Conference criteria for MCS (13) and VS. Subjects were excluded if they were ventilator-dependent, had a refractory seizure disorder, or had an MRI-incompatible device. All subjects received a neurologic examination, including a Coma Recovery Scale––Revised (14) assessment, on initial evaluation. The clinical evaluation was made at least four times in 2 weeks before the ASL study, and the last assessment was done 3 to 7 days before the ASL study. The diagnosis of VS and MCS was made when the last three assessments were equal. All neurologic examinations were done by YY and HJ, who had 10 and 18 years of experience as neurosurgeons, respectively. Healthy control subjects, aged from 20 to 70 years, were recruited by advertisement. Study exclusion criteria for healthy volunteers included contraindications to MRI; pregnancy, major head trauma, abnormal structural MRI, and the presence of other neurological diseases. Excluded medications included psychoactive medications, nitrates, and warfarin or other drugs that may affect CBF.

### MRI Protocol

Patients and control subjects underwent structural and functional imaging studies. All image data were acquired on a 3.0-Tesla MRI scanner system (GE Medical Systems, Milwaukee, WI, USA). Structure imaging included 3D-T1-weighted and T2-weighted images. ASL sequences were obtained during the awake resting state. Patient and control ASL sequences with significant motion degradation were excluded from analysis.

The raw ASL images were acquired twice using 3D pcASL sequences with a post-labeled delay (PLD) time of 1.5 or 2.5 s (15–17). Images were acquired with the following parameters: 512 sampling points on eight spirals, spatial resolution = 3.64 mm, TR = 4,590 (PLD = 1.5 s)/5,285 ms (PLD = 2.5 s), TE = 10.5 ms, slice thickness = 4 mm, number of slices = 36, acquisition time = 4:29 (PLD = 1.5 s)/5:09 (PLD = 2.5 s) minutes, field of view (FOV) = 24 cm, and number of excitations (NEX) = 3. A high-resolution volumetric T1-weighted sequence of the whole brain were acquired with the following parameters: TR = 8.2 ms, TE = 3.2 ms, TI = 450 ms, FOV = 24 cm, slice thickness = 1 mm, number of slices = 156, acquisition time = 4:08 min, matrix = 256 × 256, and NEX = 1. The CBF map was calculated with commercial software on the GE AW workstation. The psASL has been demonstrated to be both precise and reliable compared with the gold standard 15O-water PET (18).

### Analysis Methods

The CBF maps were registered to 3D-T1WI structure imaging, and the 3D-T1WI images were used for image registration and normalization into a standardized space (Montreal Neurological Institute template, MNI space) within the Statistical Parametric Mapping (SPM8)^1^ on MatLab 7 (MathWorks, Natick, MA, USA). Anatomical regions-of-interest (ROIs) were generated from WFU Pickatlas (Wake Forest University).^2^ Ten typical combined bilateral ROIs were selected on the basis of a literature review of previous ASL studies and included the caudate, putamen (PUT), thalamus (THAL), anterior cingulate gyrus, medial frontal cortex, middle frontal cortex, superior temporal gyrus, posterior cingulate, parietal cortex, and occipital pole (19). Next, multiple brain ROIs of ASL CBF map were automatically separated based on the Automated Anatomical Labeling-116 (AAL-116) brain template (20) and the bilateral Brodmann’s template, as AAL and Brodmann’s templates are generally used in previous function imaging studies. On the AAL temple, we choose ROIs on left and right separately. We decided to omit cerebellar values from our analysis, given that imaging coverage of the cerebellum using the pulsed-continuous method is variable and subject to systematic error (21).

The average CBF was calculated for each structure for both control subjects and patients. Then the average CBF value of every brain region was compared between the MCS and VS groups with *t*-tests.

For a global analysis of CBF, GM was separated within the WFU Pickatlas template. Two deep white matter (WM) masks were manually drawn to avoid GM–WM contamination (18, 22). This was done by selecting sphere-shaped voxels with a 10-mm radius at the right and left centrum ovale.

Differences in CBF in GM and WM between patients and controls were calculated using a two-sample *t*-test assuming unequal variances.

## Results

### Demographics

First, we visually checked the quality of the CBF map, and the 3D high-resolution brain structural images for all the patients using the methods on reference (23). Skull distortions in TBI patients can make difficult to normalize neuroimaging data. After carefully visually inspecting the brain images, we excluded 13 patients from the recruited patients for head motion, brain deformation, artifact, or severe hydrocephalus.

Fifteen patients with VS ranged in age from 23 to 71 years, and included 5 women and 10 men. Eight patients met the MCS criteria, and they ranged in age from 23 to 61 years, and included one woman and seven men. Etiologies included traumatic brain injury, stroke, and hypoxic-ischemic encephalopathy (Table 1). Patients were within an interval from injury to evaluation ranging from 1 month to 47 months. Twenty-seven healthy volunteers (13 women and 14 men) were identified, ranging in age from 28 to 56 years. The differences of etiology, age, sex, and duration of disease were not statistically significant.

**Table 1:** Patient data.

Case no.	Diagnosis according to CRS-R	CRS-R total score (and subscores^a^)	Etiology	Lesions^b^ on T1WI L/R/B/N	Duration (months)
1	MCS	17 (3, 4, 5, 1, 1, 3)	T	N	1
2	MCS	10 (2, 3, 2, 1, 0, 2)	n-T (anoxia)	B	3
3	MCS	9 (1, 3, 2, 1, 0, 2)	T	N	6
4	MCS	11 (1, 3, 4, 1, 0, 2)	T	L	2
5	MCS	7 (1, 0, 3, 1, 0, 2)	T	L	3
6	MCS	11 (3, 3, 2, 1, 0, 2)	T	N	7
7	MCS	9 (1, 3, 2, 1, 0, 2)	T	R	2
8	MCS	8 (1, 1, 3, 1, 0, 2)	T	R	47
9	VS	7 (1, 1, 2, 1, 0, 2)	T	L	1
10	VS	7 (1, 1, 2, 1, 0, 2)	n-T (stroke)	L	1
11	VS	5 (0, 0, 2, 1, 0, 2)	n-T (anoxia)	B	9
12	VS	6 (1, 0, 2, 1, 0, 2)	n-T (anoxia)	B	4
13	VS	7 (1, 1, 2, 1, 0, 2)	n-T (stroke)	R	6
14	VS	7 (1, 1, 2, 1, 0, 2)	n-T (anoxia)	B	2
15	VS	3 (1, 0, 1, 1, 0, 0)	T	R	6
16	VS	5 (1, 1, 2, 1, 0, 0)	T	L	1
17	VS	7 (1, 1, 2, 1, 0, 2)	T	L	3
18	VS	5 (0, 0, 2, 1, 0, 2)	n-T (anoxia)	B	2
19	VS	6 (1, 0, 2, 1, 0, 2)	n-T (anoxia)	B	8
20	VS	6 (1, 0, 1, 2, 0, 2)	T	R	4
21	VS	6 (1, 1, 2, 1, 0, 1)	T	R	3
22	VS	6 (1, 0, 2, 1, 0, 2)	T	R	17
23	VS	6 (1, 1, 1, 1, 0, 2)	T	L	3

*T, traumatic; n-T, non-traumatic*.

*^a^Coma Recovery Score––Revised (CRS-R) subscores are in the following order: auditory, visual, motor, verbal, communication, and arousal*.

*^b^Lesions on T1WI: the L, R, B, and N equal left, right, bilateral, and none lesions on the cerebral hemispheres*.

### CBF Differences: Patients vs. Control Subjects

**Figure 1:**
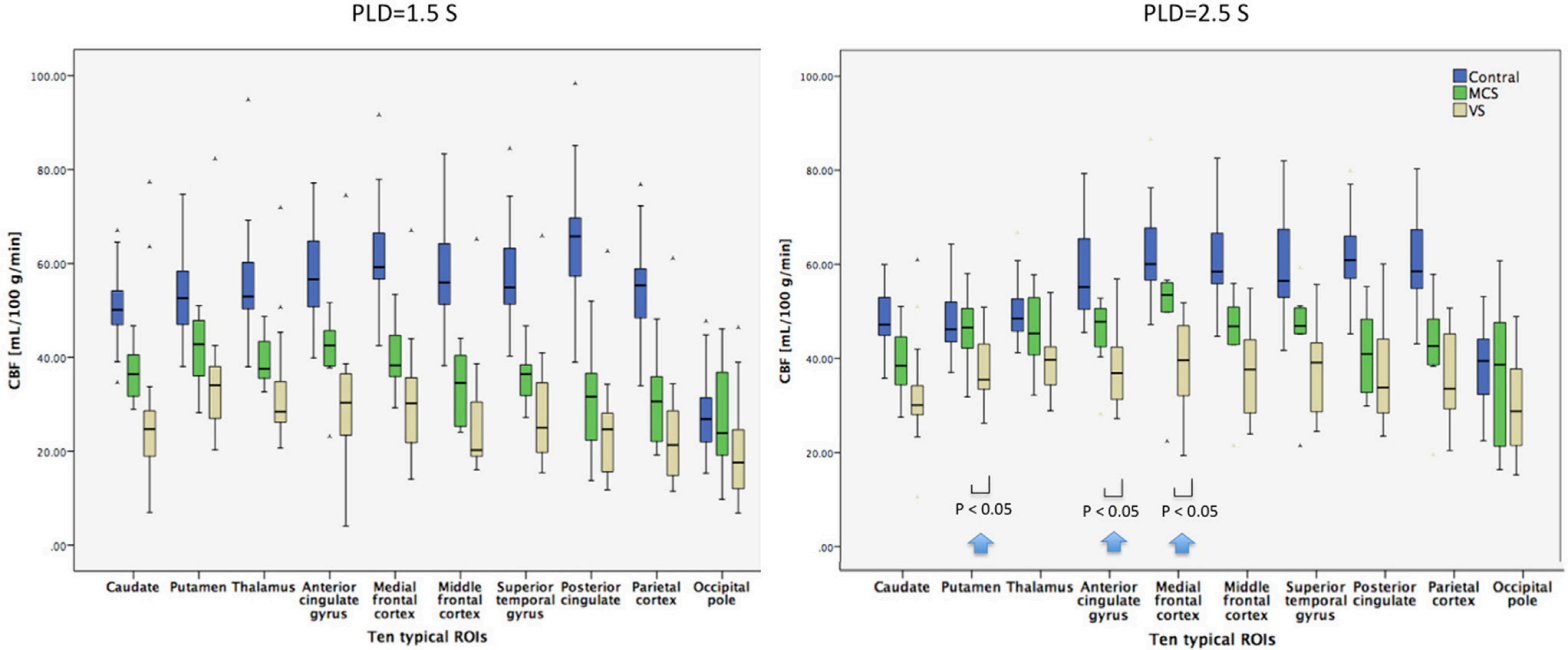
Box plots displaying 25, median value, 75 (box), and 95% (whiskers) of cerebral blood flow (CBF) value distributions in the 10 typical regions of interest (ROIs) of healthy control subjects, minimally conscious state (MCS) patients, and vegetative state (VS) patients. Compared with VS patients, the regional mean CBF with post-labeled delay (PLD) 1.5 s for MCS patients had a pattern of relatively decreased CBF in most ROIs, but not significantly. As the PLD changed to 2.5 s, the regional mean CBF for MCS had a pattern of significantly increased CBF in the regions including the putamen, anterior cingulate gyrus, and medial frontal cortex (*P* < 0.05) (as shown by arrows).

The 10 typical ROIs CBF of the control subjects ranged from 15.3 to 98.3 (median value 54.5) mL/100 g/min on PLD 1.5 s and from 22.4 to 86.5 mL/100 g/min (median value 30.2) on PLD 2.5 s (Figure 1). The 10 typical ROIs CBF of the MCS and VS patients showed greater variability and ranged from 4.1 to 83.3 (median value 54.2) mL/100 g/min on PLD 1.5 s and from 7.8 to 60.9 (median value 39.8) mL/100 g/min on PLD 2.5 s.

#### PLD 1.5 vs. 2.5 s

The mean CBF for control subjects and patients on PLD 1.5 and 2.5 s was calculated for each of the 10 ROIs. For most regions in the control subjects, mean CBF changed less than did PLD from 1.5 to 2.5 s (Figure 1). On the contrary, mean CBF increased for most ROIs of patients as PLD changed from 1.5 to 2.5 s. In general, the difference of mean CBF between MCS patients and control subjects was smaller on PLD 2.5 s than on 1.5 s.

#### Ten Typical ROIs

The mean CBF for control subjects was significantly higher than that for patients for nine ROIs on PLD 1.5 s, except the occipital pole. CBF for control subjects was significantly higher than that for patients for all 10 ROIs on PLD 2.5 s.

#### AAL-116 Template

The mean CBF for control subjects was significantly higher than that for patients for every ROI on PLD 1.5 s, whereas the mean CBF for control subjects was significantly higher than that for patients for every ROI on PLD 2.5 s except bilateral pallidum (PAL).

#### Bilateral Brodmann’s Template

The mean CBF for control subjects was significantly higher than that for patients for every ROI on PLD 1.5 s except the lateral globus pallidus, medial globus pallidus, and hypothalamus, whereas the mean CBF for control subjects was significantly higher than that for patients for every ROI on PLD 2.5 s except Brodmann’s area 27, the hypothalamus, medial geniculum body, PUT, ventral lateral nucleus, lateral posterior nucleus, lateral globus pallidus, ventral posterior medial nucleus, medial globus pallidus, ventral posterior lateral nucleus, lateral geniculum body, and subthalamic nucleus.

#### GM and WM

The mean CBF of GM and WM for control subjects was significantly higher than that for patients on PLD 1.5 and 2.5 s.

### CBF Differences: MCS vs. VS

#### Ten Typical ROIs

Compared with VS patients, the regional mean CBF with PLD 1.5 s for MCS had a pattern of relatively decreased CBF in most ROIs, but not significantly. As the PLD changed to 2.5 s; however, the regional mean CBF for MCS had a pattern of significantly increased CBF in the regions including PUT, anterior cingulate gyrus, and medial frontal cortex (*P* = 0.0164, 0.0497, and 0.0288) (Figure 1).

#### AAL-116 Template

**Figure 2:**
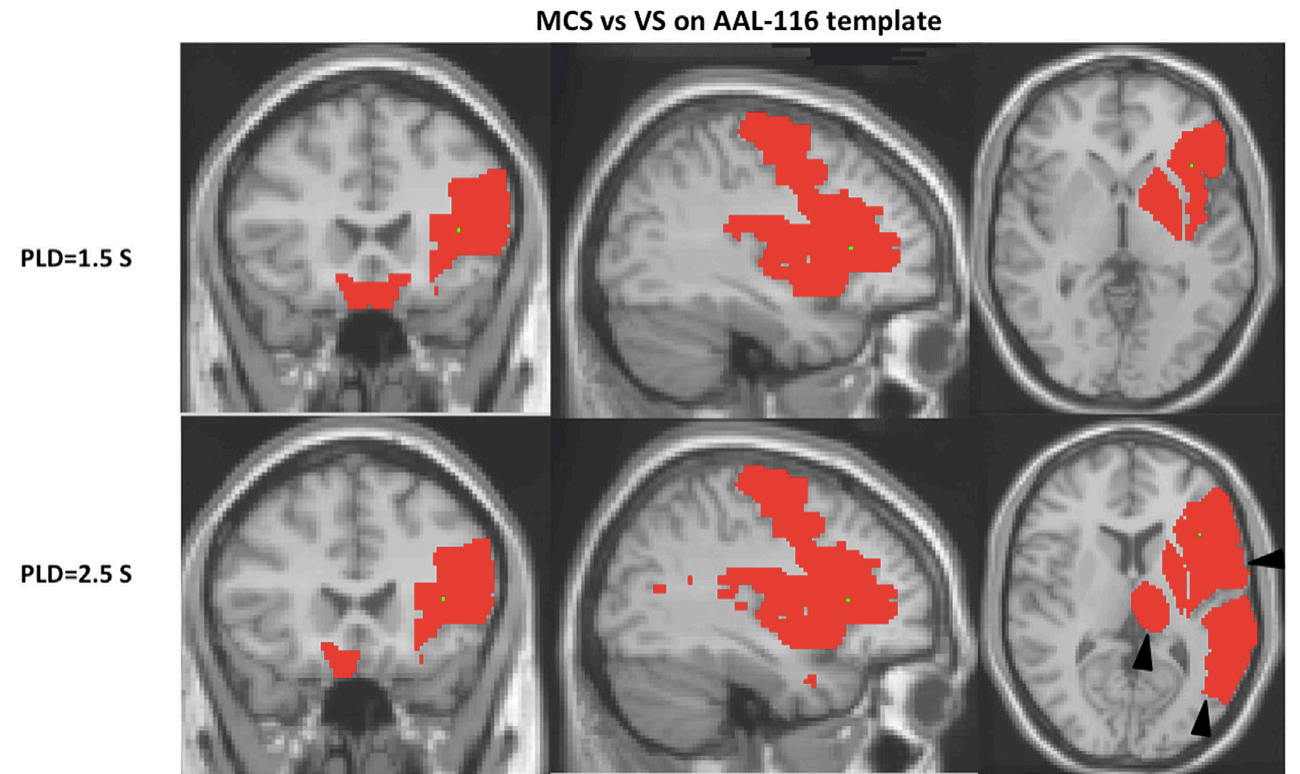
On the Automated Anatomical Labeling-116 (AAL-116) template, a left-lateralized pattern is observed. With regard to the cerebral blood flow (CBF) of single AAL regions separating vegetative state (VS) from minimally conscious state (MCS), the MCS regions were generally left-lateralized. CBF with post-labeled delay (PLD) 2.5 s found more regions of pattern differentiating MCS from VS than with PLD 1.5 s on AAL-116 templates, including the inferior left frontal gyrus, triangular (F3T), temporal pole: middle temporal gyrus (T2P), temporal pole: superior temporal gyrus (T1P), and thalamus (THAL) (as shown by triangles).

Compared with VS patients, the regional CBF with PLD 1.5 s for MCS had a pattern of relatively increased CBF in the regions of left inferior frontal gyrus, opercular (F3OP), precentral gyrus (PRE), rolandic operculum (RO), insula (IN), PUT, PAL, and bilateral gyrus rectus (GR) (*P* < 0.05). The regional CBF with PLD 2.5 s for MCS had a pattern of relatively increased CBF in the regions including left inferior F3OP, inferior frontal gyrus, triangular (F3T), PRE, RO, temporal pole: middle temporal gyrus (T2P), temporal pole: superior temporal gyrus (T1P), IN, PUT, THAL, and right GR (*P* < 0.05) (Figure 2; Table 2).

**Table 2:** The *P*-values of regional region-of-interest (ROI) cerebral blood flow between minimally conscious state and vegetative state patients on the Automated Anatomical Labeling-116 template with two post-labeled delay (PLD).

PLD	L/R	ROI name	*P*-value
1.5 s	L	Inferior frontal gyrus, opercular (F3OP)	0.015
1.5 s	L	Precentral gyrus (PRE)	0.047
1.5 s	L	Rolandic operculum (RO)	0.034
1.5 s	L	Insula (IN)	0.034
1.5 s	L	Putamen (PUT)	0.049
1.5 s	L	Pallidum (PAL)	0.037
1.5 s	L	Gyrus rectus (GR)	0.049
1.5 s	R	Gyrus rectus (GR)	0.048
2.5 s	L	Inferior frontal gyrus, opercular (F3OP)	0.028
2.5 s	L	Inferior frontal gyrus, triangular (F3T)	0.047
2.5 s	L	Precentral gyrus (PRE)	0.041
2.5 s	L	Rolandic operculum (RO)	0.030
2.5 s	L	Temporal pole: middle temporal gyrus (T2P)	0.014
2.5 s	L	Temporal pole: superior temporal gyrus (T1P)	0.006
2.5 s	L	Insula (IN)	0.018
2.5 s	L	Putamen (PUT)	0.039
2.5 s	L	Thalamus (THAL)	0.049
2.5 s	R	Gyrus rectus (GR)	0.045

#### Bilateral Brodmann’s Template

**Figure 3:**
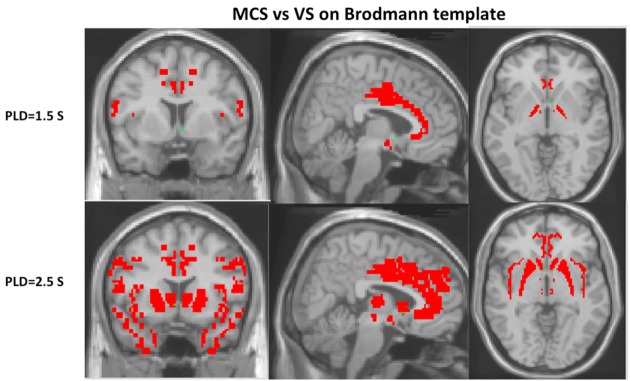
Cerebral blood flow with post-labeled delay (PLD) 2.5 s could find more regions of pattern differentiating minimally conscious state (MCS) from vegetative state (VS) than with PLD 1.5 s on Brodmann’s template, including Brodmann’s areas 9, 13, 28, 32, 33, 34, 38, medial dorsal nucleus, ventral lateral nucleus, putamen, and caudate head (*P* < 0.05).

Compared with VS patients, the regional CBF with PLD 1.5 s for MCS had a pattern of relatively increased CBF in the regions of bilateral medial globus pallidus, Brodmann’s area 24, Brodmann’s area 44, hypothalamus, and optic tract (*P* < 0.05). The regional CBF with PLD 2.5 s for MCS had a pattern of relatively increased CBF in the regions including Brodmann’s area 9, Brodmann’s area 13, Brodmann’s area 24, Brodmann’s area 28, Brodmann’s area 32, Brodmann’s area 33, Brodmann’s area 34, Brodmann’s area 38, Brodmann’s area 44, medial dorsal nucleus, ventral lateral nucleus, PUT, hypothalamus, and caudate head (*P* < 0.05) (Figure 3).

#### GM and WM

**Figure 4:**
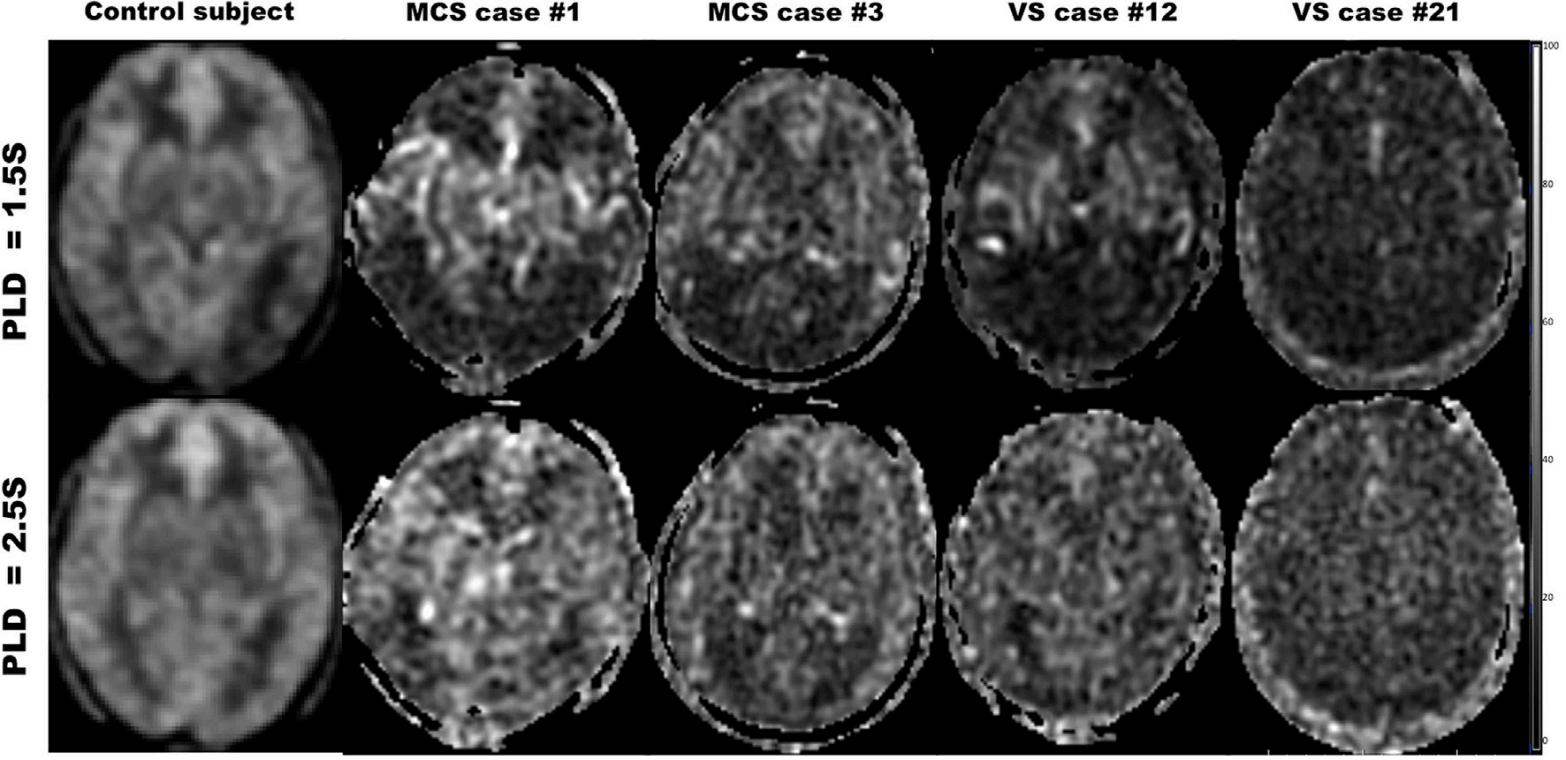
Cerebral blood flow maps with post-labeled delay (PLD) 1.5 and 2.5 s for the healthy control subject, minimally conscious state (MCS) patients, and vegetative state (VS) patients.

The mean CBF of GM or WM for MCS was not significantly higher than that for VS on PLD 1.5 or 2.5 s (Figure 4).

## Discussion

Our study demonstrates globally reduced ASL-measured CBF in MCS or VS patents compared with normal controls, particularly with PLD 1.5 s. Compared with VS, the regional CBF for MCS had a pattern of significantly increased CBF in the regions including the PUT, anterior cingulate gyrus, and medial frontal cortex with PLD 2.5 s, within 10 typical ROIs., In addition, a left-lateralized pattern is observed to differentiate MCS from VS on AAL-116 template. Moreover, regional CBF with PLD 2.5 s could find more regions of pattern differentiating MCS from VS than with PLD 1.5 s, except for globus pallidus.

This study found globally reduced ASL-measured CBF in MCS or VS patients compared with normal controls, particularly with PLD 1.5 s, which were similar with previous studies with ASL (12) and PET (24). Our finding of decreased relative blood flow to the medial frontal cortex and frontal areas in our MCS or VS patients is consistent with previous work highlighting the vulnerability of the anterior forebrain after severe brain injury. Various brain injuries ultimately disrupt the corticostriatopallidal–thalamocortical projection system’s ability to modulate the anterior forebrain (25). In this study, the CBF of the area of the PUT, PAL, and THAL was higher in MSC than in VS.

Preserved relative blood flow to the medial frontal cortex, left temporal–parietal areas, and left THAL, which were parts of the default mode network (DMN), appears to be another defining characteristic of our sample of MCS patients, consistent with emerging functional MRI (fMRI) research. The DMN consists of a set of regions, including the medial prefrontal cortex, precuneous/posterior cingulate, bilateral temporal–parietal areas, and THAL, which are more active at rest than during attention-demanding tasks (26). The DMN has been proposed as the substrate for consciousness (27). It was also reported that fMRI connectivity to the precuneous/posterior cingulate region could differentiate MCS patients from unconscious patients (28). It remains to be investigated whether MCS and VS can be differentiated by different patterns of regional CBF as measured by ASL.

On the AAL-116 template, as we choose the ROIs left and right separately, a left-lateralized pattern is observed. With regard to the CBF of single AAL regions, separating VS from MCS, the MCS regions were generally left-lateralized. Rosazza et al. found that the differences between VS and MCS patients were stronger for the left than the right hemisphere for resting-state fMRI and 18F-fluorodeoxyglucose (FDG)-PET, and that the integrity of the left hemisphere is predictive of better clinical status (29). Based on behavioral observation of patients, it has also been hypothesized that the functional preservation of the left hemispheric function has greater impact on level of consciousness than the right one (e.g., Serafetinides et al. (30), Glosser et al. (31), and Meador et al. (32)). The study based on VBM found that GM atrophy in disorders of consciousness appeared to be mostly left lateralized (33). Left-lateralized atrophy in disorders of consciousness also made a tentative association with severely impaired language processing (34, 35). Cerebral atrophy has been shown to be associated with globally decreased blood flow (36, 37). However, as the evidence for left lateralization of the neural foundations of consciousness remains controversial, further confirmation of this finding is required.

Different PLDs of pcASL had considerable influence on MCS and VS. CBF with PLD 2.5 s could find more regions of pattern differentiating MCS from VS than with PLD 1.5 s on the AAL-116 or Brodmann’s template, including inferior left F3T, T2P, T1P, THAL, Brodmann’s areas 9, 13, 28, 32, 33, 34, 38, medial dorsal nucleus, ventral lateral nucleus, PUT, and caudate head. On one hand, longer PLD means more blood flows into the regional brain (16, 38, 39). On the other hand, longer PLD will decrease the SNR of ASL, as the magnetically labeled water in ASL only has 1–3 s of half-life. Moreover, the spins are exposed to the presence of a substantial amount of iron in the basal ganglia regions, resulting in further T1 shortening (40) and lower apparent CBF values with longer PLD. The apparent regional ASL CBF was the result of the balance among the aforementioned three aspects at least (16). For MCS and VS patients, longer PLD made the regional CBF higher than short PLD, especially for MCS. Therefore, CBF with PLD 2.5 s could find more regions of pattern differentiating MCS from VS than with PLD 1.5 s (Figure 4). However, as for globus pallidus, the substantial amount of iron could make the apparent CBF lower on PLD 2.5 s. Thus, only CBF with PLD 1.5 s could find the difference of globus pallidus CBF between MSC and VS (Figure 4).

One of the major limitations in our study was motion artifact, a common problem in ASL. Although our methods selected for data not significantly motion-degraded, even slight motion artifact disturbed their CBF values. Another consideration in interpreting our results is the inter-subject variation, such as age and gender. Previous work has demonstrated that older subjects have significantly decreased CBF compared with younger counterparts, especially to the frontal cortex (36, 41). Likewise, women overall have increased global CBF, approximately 13% higher than men (36). Moreover, our patients were studied in 1 to 47 months after brain injury, possibly introducing serious bias in the patient selection. There is some consensus that the late subacute phase (days 14–20) may be optimal for imaging. By this time, brain edema has subsided, and many critical decisions in medical and ethical management have been made (42). However, the short time (i.e., 1–2 months) could imply uncertainty or fluctuation of clinical diagnosis, since these patients at this time could have high probability of ongoing clinical evolution. More investigation on the use of functional imaging in general, including ASL, is needed at this pivotal stage in medical decision-making. Finally, the sample size was very different among patients in an MCS (*n* = 8), patients with VS (*n* = 15), and healthy control subjects (*n* = 23). The different sample sizes between MCS and VS patients might explain the lack of statistical significance in some results.

## Conclusion

We identified a selective reduction of CBF within specific brain regions in VS patients compared with MCS patients. Therefore, MCS might be differentiated from VS by different ranges of regional CBF as measured by ASL. Multi-PLD ASL may serve as an adjunctive method to separate MCS from VS and assess functional reserve in patients recovering from severe brain injuries. Because of its advantages of speed and ease of acquisition and its ability to provide precise quantitative CBF, ASL could be used in longitudinal assessments of patients with severe brain injuries.

## Ethics Statement

All procedures performed in the studies involving human participants were in accordance with the ethical standards of the institutional review boards of PLA Army General Hospital and with the 1964 Helsinki Declaration and its later amendments or comparable ethical standards. The datasets generated during and/or analyzed during the current study are available from the corresponding author on reasonable request.

## Author Note

Statistical Analysis Conducted by Dr. Bing Wu, MD, PLA Army General Hospital.

## Author Contributions

BW and YY contributed to the study concept and design, and analysis and interpretation of data. SZ, WW, ZW, and GH contributed to the acquisition, analysis, and interpretation of data. JH and XW contributed to the study concept and design, and critical revision of the manuscript.

## Conflict of Interest Statement

The authors declare that the research was conducted in the absence of any commercial or financial relationships that could be construed as a potential conflict of interest.

## Footnotes

^1^http://www.fil.ion.ucl.ac.uk/spm/software/spm8/.

^2^http://fmri.wfubmc.edu/cms/software.

## Abbreviations

ASL, arterial spin labeling; CBF, cerebral blood flow; CRS-R, Coma Recovery Score––Revised; DMN, default mode network; GM, gray matter; MCS, minimally conscious state; VS, vegetative state; WM, white matter.
